# BAP1 Loss, Nuclear Grading, and Nonepithelioid Features in the Diagnosis of Mesothelioma in Italy: Nevermore without the Pathology Report

**DOI:** 10.3390/jpm14040394

**Published:** 2024-04-08

**Authors:** Giulio Rossi, Luisella Righi, Francesca Barbisan, Marcello Tiseo, Paolo Spagnolo, Federica Grosso, Pasquale Pisapia, Umberto Malapelle, Marika Sculco, Irma Dianzani, Laura Abate-Daga, Maria Cristina Davolio, Giovanni Luca Ceresoli, Domenico Galetta, Giulia Pasello, Silvia Novello, Paolo Bironzo

**Affiliations:** 1 Pathology Unit, Services Area, Fondazione Poliambulanza Hospital Institute, Via Bissolati 57, 25124 Brescia, Italy; giurossi68@gmail.com; 2 Fondazione FONICAP, Via Locchi, 26, 37124 Verona, Italy; 3 Department of Oncology, University of Turin, San Luigi Hospital, 10043 Orbassano, Italy; luisella.righi@unito.it (L.R.); silvia.novello@unito.it (S.N.); paolo.bironzo@unito.it (P.B.); 4 Pathological Anatomy Institute, Polytechnic University of Marche Region, 60126 Ancona, Italy; francesca.barbisan@ospedaliriuniti.marche.it; 5 Department of Medicine and Surgery, University of Parma and Medical Oncology Unit, University Hospital of Parma, 43126 Parma, Italy; marcello.tiseo@unipr.it; 6 Respiratory Disease Unit, Department of Cardiac, Thoracic, Vascular Sciences and Public Health, University of Padova, 35128 Padova, Italy; paolo.spagnolo@unipd.it; 7 Mesothelioma Unit, AO SS. Antonio e Biagio e Cesare Arrigo, 15121 Alessandria, Italy; federica.grosso@ospedale.al.it; 8 Department of Public Health, Federico II University of Naples, Via Sergio Pansini 5, 80131 Naples, Italy; umberto.malapelle@unina.it; 9 Department of Health Sciences, Università del Piemonte Orientale, 28100 Novara, Italy; marika.sculco@uniupo.it (M.S.); irma.dianzani@med.uniupo.it (I.D.); 10 TU.TO.R. Tumori Toracici Rari, Patient Advocacy, 20123 Milan, Italy; laura.abatedaga@gmail.com; 11 Legal Medicine and Risk Management Department, Azienda Unità Sanitaria Locale di Modena, Strada Martiniana, 21, 41126 Modena, Italy; m.davolio@ausl.mo.it; 12 Medical Oncology, Humanitas Gavazzeni Clinic, 24125 Bergamo, Italy; giovanniluca.ceresoli@gmail.com; 13 Medical Thoracic Oncology Unit, IRCCS Istituto Tumori “Giovanni Paolo II”, 70124 Bari, Italy; galetta@oncologico.bari.it; 14 Medical Oncology 2, Veneto Institute of Oncology IOV-IRCCS, 35128 Padua, Italy; giulia.pasello@unipd.it; 15 Department of Surgery, Oncology and Gastroenterology, University of Padua, 35128 Padua, Italy

**Keywords:** mesothelioma, pleura, BAP1, sarcomatoid, epithelioid, nuclear grading, immunotherapy

## Abstract

The pathologic diagnosis of pleural mesothelioma is generally based on international guidelines, but no compulsory points based on different drugs approvals in different European countries are required to be reported. According to the last (2021) edition of the World Health Organization classification of pleural tumors, the nuclear grade of epithelioid-type mesothelioma should be always inserted in the pathologic report, while the presence of BRCA-associated protein-1 (BAP1) (clone C4) loss and a statement on the presence of the sarcomatoid/nonepithelioid component are fundamental for both a screening of patients with suspected *BAP1* tumor predisposition syndrome and the eligibility to perform first-line immunotherapy at least in some countries. Several Italian experts on pleural mesothelioma who are deeply involved in national scientific societies or dedicated working groups supported by patient associations agreed that the pathology report of mesothelioma of the pleura should always include the nuclear grade in the epithelioid histology, which is an overt statement on the presence of sarcomatoid components (at least 1%, in agreement with the last classification of pleural mesothelioma) and the presence of BAP1 loss (BAP1-deficient mesothelioma) or not (BAP1-retained mesothelioma) in order to screen patients possibly harboring *BAP1* tumor predisposition syndrome. This review aims to summarize the most recent data on these three important elements to provide evidence regarding the possible precision needs for mesothelioma.

## 1. Introduction

Among the pathology community, some malignancies are well characterized by detailed checklist/template-based diagnostic reports, thus guiding pathologists in correctly defining clinically fruitful tumor characteristics, either with respect to morphology, the expression of immunohistochemical markers, and/or molecular features (e.g., breast cancer and gastrointestinal stromal tumor/GIST). At the moment, there are no definitive rules in many other oncologic fields with respect to reporting, including pleural mesothelioma (PM), except for some international proposals (i.e., from the College of American Pathologists, CAP, or the International Collaboration on Cancer Reporting, ICCR). The diagnosis of PM is generally premised on careful morphologic examination on well contrasted hematoxylin–eosin-stained slides from pleural samples and supported by ancillary techniques defining the mesothelial differentiation of the neoplastic growth (demonstrated in the epithelioid subtype by at least two positive mesothelial markers and two negative carcinoma markers) and the cell malignant nature (particularly when lacking an overt invasion of soft tissues, the parietal pleuro/chest wall, or lung parenchyma) [1,2]. This approach follows both international guidelines [1,3] and the last World Health Organization (WHO) classification [4], where particular attention is paid to morphological features and immunohistochemical profiling. 

BRCA-associated protein-1 (*BAP1*) is a gene that maps to human chromosome 3p21.3 encoding for the BAP1 protein, which is a ubiquitin carboxy-terminal hydrolase (UCH) and member of the deubiquitylase (DUB) protein family that can be found both in the nucleus and in the cytoplasm of almost all cells [5]. The *BAP1* gene was identified and named in 1998 as a powerful tumor suppressor gene [5], while in 2011 its association with germline and somatic carcinogenesis of mesothelioma was defined [6,7]. Since then, BAP1 immunohistochemical testing has become an important step of the pathologic diagnosis of PM. BAP1 status in PM histologic examination can inform diagnosis, prognosis, and possibily cancer prevention in those patients with hereditary *BAP1* mutations, and it is a main focus of clinical research for personalized treatments [8,9].

Another important issue of the PM diagnosis is the assessment of the tumor grading score according to the last WHO classification parameters, namely nuclear grade, mitosis, and necrosis. While the previous Kadota’s grading system utilized a three-tier approach based on nuclear atypia and mitotic count [10], a two-tier system of high and low grade is now favored and proposed [2].

Lastly, based on recent clinical data on immunotherapy, the careful identification and reporting of a detailed subtype has become fundamental for selecting patients for treatment, at least in some countries such as Italy. 

The present review tries to summarize the recent important evidence regarding these issues in PM diagnosis.

## 2. Role of BAP1 Protein Loss in the Diagnosis of Mesothelioma

In the last 2021 WHO classification [4], the term malignant was discouraged in the report of PM, as it is now considered malignant by definition. The PM diagnostic workflow is still largely based on the careful morphological examination of hematoxylin–eosin-stained slides from pleural samples to firstly assess the histologic subtypes: epithelioid, sarcomatoid, or biphasic. The correct recognition and classification of the specific subtype has an important prognostic role [11] and is based on architectural growth patterns, and cytologic, and stromal features often suggestive of the malignant nature of the proliferation. As a second step, the recognition/confirmation of mesothelial origin by immunohistochemistry (IHC) is mandatory [12,13,14]. As guidelines recommend, the use of at least two mesothelial positive markers and two other-nature negative markers guarantees specific differential diagnosis [1]. Recently, larger immunohistochemical panels have been proposed to improve sensitivity and specificity: while individual immunohistochemical markers showed ≈50% sensitivity, the use of multiple markers makes it possible to increase sensitivity while maintaining high specificity [12,15].

**Figure 1:**
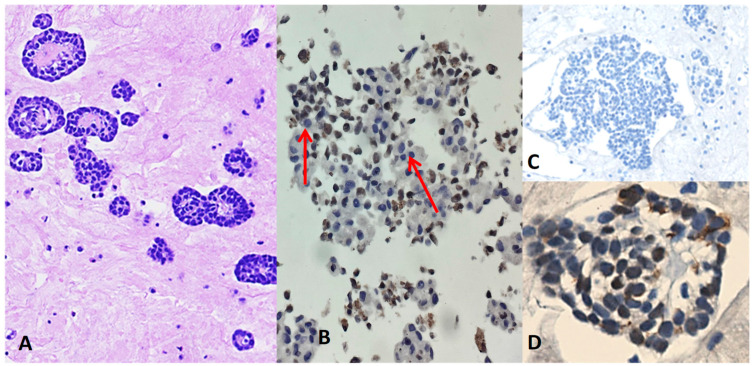
A case of diffuse epithelioid mesothelioma in an 87-year-old woman with left-sided recurrent pleural effusion. Cytology shows a well-differentiated mesothelial growth pattern with tubule–papillary structures, no necrosis, and no cell atypia in absence of mitosis, thus evidencing a low nuclear grade ((**A**), hematoxylin –eosin stain, 100×). Mesothelial neoplastic cells demonstrated loss of BAP1 expression ((**B**), immunohistochemistry, clone C4 (400×): note the unstained gray-blue nuclei with an aberrant and aspecific/insignificant granular brownish cytoplasm). Lack of expression for the most specific and sensitive nonmesothelial markers, namely claudin-4 ((**C**), immunohistochemistry, 200×) and positive nuclear staining with WT1 ((**D**), immunohistochemistry, 200×). No biopsies will be performed in light of a consistent diagnosis on cytology, the fragile condition of the elderly lady, and comorbidities. Nonepithelioid component is absent (0%).

**Figure 2:**
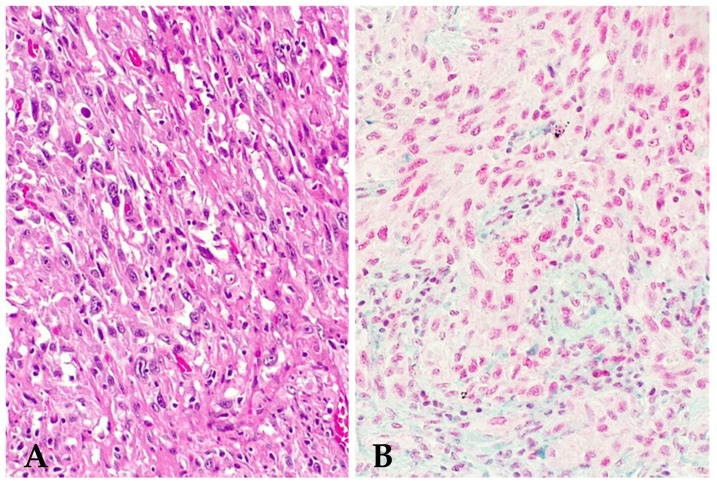
Sarcomatoid mesothelioma, high grade, with prominent spindle cell morphology without heterologous elements ((**A**), hematoxylin –eosin stain, 200×), homogeneously retaining BAP1 expression ((**B**), immunohistochemistry, clone C4, 200×).

**Figure 3:**
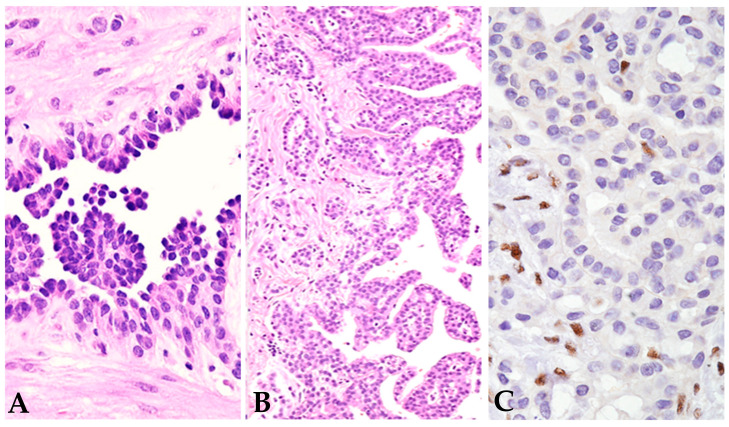
Epithelioid mesothelioma with favorable tubulopapillary pattern ((**A**,**B**), hematoxylin –eosin stain, 200×, 10×), low nuclear grade, and homogeneous loss of BAP1 ((**C**), immunohistochemistry, clone C4, 400×: some scattered positive normal endothelial cells served as internal control). The patient is a young lady with *BAP1* tumor predisposition syndrome.

Furthermore, among methods aimed at confirming the malignant nature of the mesothelial proliferation, IHC demonstrating nuclear BAP1 loss and/or the aberrant expression of Methyl-Tio-Adenosin Phosphorylase (MTAP) proteins are the most useful, but even *CDKN2A* gene deletion by fluorescent in situ hybridization, the molecular demonstration of DNA methylation profiling, or gene expression analysis using the NanoString System have been proposed with robust results [16,17,18,19,20,21]. In 2015, Cigognetti et al. [16] first demonstrated that a loss of BAP1 IHC expression is a consistent marker of the neoplastic nature in differential diagnosis with reactive mesothelial proliferation. This nuclear IHC marker is a reliable tool also in effusion cytology (Figure 1) [22]. BAP1 loss is very homogeneous in neoplastic PM cells in the vast majority of cases (>70% of analyzed cases), although heterogenous patterns or even aberrant cytoplasmic staining have been rarely reported [17]. A recent study by De Rienzo et al. [23] on a large cohort of 596 mesothelioma patients examined the associations of BAP1 staining patterns with clinical and molecular features to assess the impact of *BAP1* mutation on PM biology. In detail, four BAP1 staining patterns were described: single nuclear staining positivity (36%), single cytoplasmic staining positivity (25%), single absent staining (12%), and combinations of these staining patterns (27%). This study confirmed prior reports that nuclear BAP1 expression is more frequently associated with wild-type *BAP1* and sarcomatoid histology. Furthermore, the authors reported that BAP1 staining patterns were significantly associated (*p* < 0.001) with *BAP1* gene expression, PM histologic subtypes, molecular clusters, and markers of epithelial-to-mesenchymal transition. In epithelioid subtypes, BAP1 loss reached 62% in frequency [24], while in nonepithelioid or sarcomatoid histology, the BAP1 was generally retained, and its loss characterized less than 50% of cases [17] (Figure 2). BAP1 nuclear loss by IHC is very sensitive to detect *BAP1* biallelic inactivation, because approximately all pathogenic *BAP1* mutations are either mutations abolishing the expression of the BAP1 protein or truncating mutations causing deletion of the nuclear translocation signal, or impairing the BAP1 deubiquitylating activity, which is required for BAP1 protein nuclear translocation [25]. These data allow us to conclude that BAP1 protein loss has a 100% specificity in the distinction between benign and malignant mesothelial proliferations, particularly in epithelioid histology and including in situ mesothelioma [4] (Figure 3). BAP1 loss is also very useful in the differentiation of PM from metastatic cancers from various sites to the pleura that generally retain the protein in the nuclei [12,26,27]. 

In contrast to other markers, BAP1 loss has been analyzed in terms of patient’s prognosis with controversial results: Cantini et al. [28] reported a median OS time of 14.8 months (95% CI: 10.7–29.3) and 18.1 months (95% CI: 11.2–25.8) for negative and positive BAP1 expression, respectively (*p* = 0.2). In another study by Forest et al. [29] BAP1 loss was associated with statistically significant longer survival in patients with PM (*p* = 0.034). Finally, in the Ramucirumab Mesothelioma clinical trial (RAMES) [30], mutation of the *BAP1* gene was associated with a prolonged median progression-free survival (mPFS) in those patients treated with platinum/pemetrexed regimens (*p* = 0.04) (Table 1).

Lastly, and most importantly, BAP1 loss by immunohistochemistry is the cheapest, most rapid (about 5 EUR × test and 1 working day in a conventional pathology lab of a secondary hospital), and most reproducible method to screen patients with PM possibly harboring the syndromic disease, specifically when integrating its loss with clinical data (see below).

For all the aforementioned information related to BAP1 expression, we strongly recommended to add BAP1 staining/loss result in PM pathologic reports.

## 3. Role of BAP1 Protein Loss in the Diagnosis of BAP1 Syndrome

Germline pathogenic mutations in the tumor suppressor gene BRCA1-associated protein-1 (*BAP1*) lead to *BAP1* tumor predisposition syndrome (*BAP1*-TPDS) [6], which is characterized by high susceptibility to several tumor types, mainly melanoma (especially uveal), mesothelioma, renal cell carcinoma, and basal cell carcinoma (Table 2). *BAP1*-TPDS is inherited in an autosomal-dominant fashion with a penetrance close to 100%. In general, carriers of the germline *BAP1* pathogenic variant (PV) develop tumors at a younger age as compared to patients with the same sporadic tumors [6] (Table 3). 

A recent Italian study by Sculco et al. [33] describing a ten years in the molecular diagnosis of *BAP1*-TPDS sequenced germline DNA samples from 101 individuals with suspected *BAP1*-TPDS and validated PVs by assessing *BAP1* somatic loss in matching tumor specimens. Overall, the authors found seven patients (7/101, 6.9%) carrying six different germline *BAP1* PVs, including one novel variant. Altogether, these findings have important clinical implications for the therapeutic management of *BAP1*-TPDS patients. *BAP1*-TPDS should be suspected if an individual has been diagnosed with two or more tumors of the *BAP1*-TPDS spectrum or has one *BAP1*-TPDS malignancy and a first- or second-degree relative with a tumor included in the *BAP1*-TPDS spectrum, has a personal history of two or more inactivated melanocytic tumors (BIM), or developed mesothelioma at a young age (less than 60 years) [34,35]. Sculco et al. [33] identified the loss of the *BAP1* wild-type allele in mesotheliomas but not in nonmesothelioma metachronous tumors, possibly because these tumors had a sporadic origin, or because the role of heterozygous *BAP1* PVs in carcinogenesis is tissue-dependent.

In this regard, several studies have recently shown the essential need to test and identify germline *BAP1* carriers in order to implement surveillance, which ultimately may lead to improved survival and cost savings for the healthcare system [33,36,37,38]. Thanks to the increased availability of large gene panels for tumor next generation sequencing (NGS) in clinical practice, the identification of *BAP1* carriers is expected to increase. However, for many rare cancer predisposition syndromes, no clear consensus for surveillance and management recommendations for *BAP1* carriers exists. In this context, the Clinical Guideline Working Group of the CanGene-CanVar project proposed a European collaboration of expert clinicians to develop guidelines to unify the surveillance program within Europe [34]. Germline genetic screening should be performed in the context of tumours belonging to the *BAP1* spectrum with additional supportive information (e.g., IHC BAP1loss, age of onset, and personal or family history of cancer). Considering the high somatic rate of *BAP1* alterations, germline testing should be undertaken when the variant allele frequency (VAF) is higher than 10%.

Genetic testing methodologies for the screening of patients with suspected *BAP1*-TPDS include single-gene analysis by Sanger sequencing, the NGS multigene panel test, or large-scale sequencing approaches like whole exome sequencing or whole genome sequencing. To detect the germline copy number variation (CNV), specific assays should be employed, such as the multiplex ligation-dependent probe amplification (MLPA) assay, which is very useful in the case of Sanger sequencing [33]. In the case of NGS-based methodologies, CNVs are usually suspected based on bioinformatic analysis although they must be confirmed by an alternative method such as MLPA or real-time PCR.

Cascade genetic testing should be performed when a pathogenic or likely pathogenic variant, according to ACMG/ACP guidelines, is identified, and carriers should be proposed to follow appropriate surveillance measures. Even if not generally recommended for mesothelioma, active survaillance but could be useful for other tumors of the spectrum. 

When a variant of unknown significance (VUS) is identified, cascade testing may help in the definition of pathogenicity if the variant segregates together with the occurrence of TPDS core tumors [34]. As of today, an optimal functional test to assist in VUS interpretation is still missing. Several assays have been proposed and utilized to assess some functions of variant protein in vitro, including nuclear localization, deubiquitination activity, or the effect on cell adhesion/spreading and proliferation [39,40]. However, none of these assays is considered optimal, because each investigates a single function.

On the other hand, it is possible to predict the functional impact of a variation on splicing by using an exon trapping assay through the expression of reference and variant mini genes in mammalian cells and analysis of the resultant RNA products [41]. Moreover, when fresh blood samples are available, it is possible to directly analyze the patient RNA by RT-PCR assay to detect possible abnormal-sized products.

Overall, germline *BAP1* mutations are very rare in consecutive series of mesothelioma patients [42], although approximately 6% of patients with a family history of mesothelioma and other cancers carry a pathogenic mutation [33]. Moreover, approximately 22% of all *BAP1* germline mutations carriers will develop a malignant mesothelioma during their life [43]. 

## 4. Grading System in Mesothelioma

The PM grading system according to WHO 2021 criteria ^4^ for epithelioid PM should be always inserted in the pathologic report. Nuclear grade (nuclear atypia and mitotic count) and the presence of necrosis are the main morphologic features that should be identified to assess low- or high-grade epithelioid mesotheliomas. These parameters make it possible to stratify patients’ prognosis. 

More recently, another scheme to determine the tumor grade has been proposed by Fuchs et al. [44], which not only takes into account epithelioid but also biphasic and sarcomatoid histologies. The authors proposed a mesothelioma weighted grading scheme (MWGS) ranging from 0 to 10 based on the patient’s age (≤74, >74 yrs: scores 0, 1); histologic type (epithelioid, biphasic, sarcomatoid: scores 0, 1, 2); necrosis (absent, present: scores 0, 2); mitotic count per 2 mm^2^ (≤1, 2 to 4, ≥5: scores 0, 1, 2); nuclear atypia (mild, moderate, severe: scores 0, 1, 2); and BAP1 expression (lost, retained: scores 0, 1). A total score ranging from 0 to 3 is considered as low grade, from 4 to 6 as intermediate grade, and from 7 to 10 as high grade. When applied on 369 consecutive PMs, the authors found that the median survival was 17.1, 10.1, and 4.1 months for low, intermediate, and high grades, respectively (*p* < 0.0001), and the overall survival (OS) worsened progressively with the increase in score (*p* < 0.0001). Interobserver concordance was considerable (κ = 0.588), with assessment of the nuclear grade being the most subjective parameter (κ = 0.195). The three-tiered MWGS score system was compared to the two-tiered grading system proposed in the WHO [4] that is able to predict median survival in epithelioid (median 18.0 vs. 11.3 mo, *p* = 0.003) and biphasic (16.2 vs. 4.2 mo, *p* = 0.002) but not in sarcomatoid PM (5.4 vs. 4.7 mo, *p* = 0.407). Interestingly, the WHO grading system [4] showed a significant prognostic role in mesotheliomas with BAP1 loss (median survival 18.7 vs. 10.4 mo, *p* < 0.0001) but not in cases with retained BAP1 expression (8.9 vs. 6.2 mo, *p* = 0.061). The MWGS seemed to be more effective in risk stratification, and it applies to all diffuse PMs, regardless of their histology or BAP1 status [44].

Nuclear grading on cytology has been recently proposed by Li et al. [45] when considering cytologic features with prognostic significance proposed in the 2021 WHO classification of epithelioid diffuse PM (E-DPM) [4]. The authors retrospectively assessed nuclear atypia, pleomorphic features, necrosis, and architectural patterns in 35 paired cytology and concurrent/consecutive surgical specimens of E-DPM. Agreement between pairs was determined via unweighted κ scores. The main reason for the disagreements was the sampling differences between the cytology and histology specimens. Furthermore, while mitotic counts in cytology are not reliable, and nuclear grading cannot be accurately completed, careful assessment of the nuclear atypia in cytology specimens has been proven to be reliable. The identification of pleomorphic features and necrosis was also reliable despite occasional sampling issues, while the assessment of architectural patterns seemed to more limited in cytology. Nevertheless, in cytology cases with available cell block material available (n = 23), the assessment of nuclear atypia and the presence of pleomorphic features showed perfect agreement (κ = 1.000; *p* < 0.001 each), while the presence of necrosis showed moderate agreement (κ = 0.465; *p* = 0.008), and the assessment of architectural patterns showed slight agreement (κ = 0.162; *p* = 0.15) in paired specimens.

In another recent retrospective study by Straccia et al. [46], cytological specimens from a large series of histologically proven diffuse mesothelioma patients diagnosed over 19 years were reviewed and reclassified according to the International System for Reporting Serous Fluid Cytopathology (ISRSFC) [47]. Among the 210 cases with paired cytology and biopsy, 192 (91.4%) epithelioid and 18 (8.6%) sarcomatoid subtypes were diagnosed. The cytological cases were reclassified as follows: 112 (53.4%) as malignant (MAL), 81 (38.6%) as negative for malignancy (NFM), 11 (5.2%) as suspicious for malignancy (SFM), 4 (1.9%) as atypia of undetermined significance (AUS) and 2 (0.9%) as nondiagnostic (ND). Sarcomatoid cells in the MAL category were characterized cytomorphologically by more pronounced discohesion. In comparison with the epithelioid subtype, the tumor cells appeared solitary with moderate or marked nuclear pleomorphism and irregular chromatin. The final statement of the authors highlights the importance of recognizing the cytological characteristics of the sarcomatous variant in order to suggest a precise and early diagnosis.

## 5. Sarcomatoid Histology Assessment

In the Checkmate-743 trial, first-line immunotherapy with ipilimumab and nivolumab significantly extended the overall survival of treatment-naïve patients with unresectable PM, regardless of the histological subtype (18.1 vs. 14.1 months in the chemotherapy group, with a hazard ratio (HR) of 0.73) [48,49]. Subgroup analyses revealed a significant difference in the OS gain between patients with nonepithelioid and epithelioid histology. Notably, as already seen in other trials exploring immune checkpoint inhibitors (ICIs) in solid tumors, the experimental treatment was characterized by an excess of rapid disease progressions in the first 6 to 9 months of therapy. The 3-year update of the study has confirmed these data [48]. Although the median OS with immunotherapy was similar in the two groups, the HR for survival was 0.48 (95% CI 0.34–0.68) for nonepithelioid versus 0.84 (95% CI 0.69–1.03) for epithelioid patients. 

The Italian Medicines Agency (AIFA) has recently released the long-awaited approval for ipilimumab plus nivolumab in patients with unresectable PM. Unlike the European Medicines Agency (EMA) marketing authorization, the approval and reimbursement by the Italian National Health System has been limited to patients with the nonepithelioid histologic subtype. 

Studies using whole-genome sequencing, transcriptomic, and epigenomic analysis have shown that histopathological classification only accounts for a fraction of interpatient molecular heterogeneity. Other factors, including ploidy, tumor-immune interaction, and epigenomic regulation tune the biology of the tumor and possibly its responsiveness to ICIs. Recent studies have shown that these factors may at least partially explain why patients affected by epithelioid PM with similar clinical characteristics may behave quite differently [50]. To what extent such novel tools may help in selecting patients with epithelioid PM who will derive the most benefit from ICIs and, on the contrary, those who will progress early would be of great value. As already mentioned, the current approval and reimbursement of first-line ICIs by AIFA is limited to patients affected by nonepithelioid unresectable PM. Therefore, the accurate differential diagnosis of histological subtypes becomes mandatory to not deny a potentially active treatment in patients with any sarcomatoid component. Even though open pleural biopsy is considered the gold standard diagnostic method, it is notoriously less sensitive for determining histologic subtypes, particularly with nonepithelioid tumors, than surgical pleurectomy. As macroscopically radical intent surgery is performed only in well-selected patient PMs, most patients are diagnosed, at best, with pleural biopsies during thoracoscopy. Therefore, we strongly suggest that pleural biopsies be performed at multiple sites of the involved pleura with deep sampling to obtain multiple samples recapitulating any potential disease heterogeneity. 

Clinical research is also exploring the addition of immune checkpoint inhibitors to first-line chemotherapy. The phase 3 IND-227 trial comparing platinum/pemetrexed chemotherapy to chemotherapy with the anti-programmed death protein 1 (PD-1) pembrolizumab have demonstrated a statistically significant increase in both the PFS and OS favoring the experimental treatment along with a not negligible increase in terms of the response rate [51]. However, even in this study, subgroup analysis suggests that patients with nonepithelioid PM may derive more benefit from the addition of immunotherapy. Interestingly, the addition of chemotherapy seems to avoid most early progression observed in the immunotherapy arm, as already observed in other solid tumors. The pending results of other randomized trials (BEAT-meso, NCT03762018, and DREAM3R, NCT04334759) will hopefully give more insights into the role of chemoimmunotherapy (with antiangiogenics in the BEAT-meso study) in patients with unresectable PM. 

Until that moment, the standard of care paradigm in Italy would be chemotherapy in patients with epithelioid PM and immunotherapy, with a dual checkpoint blockade in those with nonepithelioid histologies. To what extent such a restriction may hamper the accrual of patients to future international trials exploring further lines of treatment is unpredictable. On the other side, the current Italian reimbursement of the Checkmate-743 regimen may be taken as an opportunity to further define the actual real benefit of this novel combination in an extremely selected population for whom chemotherapy has always been associated with significant toxicity and less than modest benefit. 

## 6. Discussion

The clinical management and pathological diagnosis of PM is becoming challenging both for expert oncologists and pathologists. The identification of novel histologic patterns associated with distinct molecular alterations and outcome is reshaping our understanding of PM as a heterogeneous disease, thereby introducing new opportunities for diagnostic and therapeutic interventions based both on morphological and molecular findings [52]. 

Carbone et al. [25] reported a rate of incorrect diagnosis of diffuse PM of 14% in high-resource countries, increasing to 50% in developing countries, despite the continuous development of novel immunohistochemical and molecular markers. These figures should alert all the scientific community about the urgent need for more standardized diagnoses through the correct use of immunohistochemistry and molecular techniques. 

However, the diagnosis of mesothelioma has significant morphological and immunophenotypic problems that could hinder reproducibility among pathologists [53]. 

As to morphology, the recognition of a specific histotype can pose difficult differential diagnoses in some cases. Although epithelioid morphology may be the most easy to recognize, it could be comparable to several carcinomas, either primary or metastatic to the pleura, due to the variety of architectural patterns that can be seen in epithelioid PM, as well as the frequent evidence of multiple patterns in the same specimens [4]. The cytologic characteristics of epithelioid mesothelioma are also diverse, with a spectrum encompassing bland to significantly atypical neoplastic cells. Conversely, sarcomatoid carcinoma has fewer architectural patterns than epithelioid PM, and the commonly recognized patterns include fascicles, solid sheets, and infiltrating single cells. Nevertheless, the differential diagnoses with chest wall primary sarcomatous tumors or lung sarcomatoid carcinomas can be an important diagnostic challange [54].

Due to the significant histologic heterogeneity of diffuse PM, it is necessary to conduct thorough sampling and evaluation of surgical specimens for the final classification of the tumor [55]. The histologic classification of malignant mesothelioma based on biopsies is both less accurate and less prognostic than histologic classification based on surgical resection. Although sarcomatoid and biphasic diagnoses have a high specificity on biopsies, a diagnosis of epithelioid mesothelioma in the initial biopsy is not specific and could be changed to the biphasic or sarcomatoid type in up to 19.5% of cases.

The immunohistochemical profile could be equally problematic due to either aberrant expression [56] or a lack of specific markers in the most challenging cases. New single markers are continuously under investigation [57,58], and new marker panels are expected to better characterize tumor proliferation [12,15,26,59,60].

In this scenario, several molecular technologies have been proposed as ancillary tools to reach the diagnosis, but none of them seem more useful and cheaper than morphology and IHC in routine clinical practice, and their use could be advised in selected cases. The accumulation of deletions and mutations in BAP1/SETD2 (3p21), CDKN2A (9p21), and NF2 (22q12) represents the most common genomic alteration in DPM, even if not lineage-specific [52]. Other epigenetic changes have been described [61], including the methylation profile [20], gene expression analysis [21], and miRNA [62] associated to specific histotypes, although large confirmatory studies are still necessary. 

Indeed, the comparison of challenging PM cases with more expert pathologists is another good practice, especially in a historical phase of radical change in the pathology units thank to the improvement of the specimen’s traceability and diagnostic phase through fully digitalized pathology, thus likely allowing fast consultations of shared images [63]. Lastly, there is a need to standardize the diagnosis of mesothelioma among the pathology community by possibly sharing diagnostic checklists aimed at including the essential information that may properly guide therapeutic strategies. 

## 7. Conclusions and Future Directions

The pathologic diagnosis of diffuse PM should include compulsory points based on different drug approvals in different European countries and according to the last fifth edition of the WHO [4] classification of pleural tumors, such as the nuclear grade of the epithelioid-type mesothelioma, the presence of BAP1 (clone C4) protein loss, and a statement on the presence of the sarcomatoid/nonepithelioid component. The latter are fundamental for the screening of patients with suspected *BAP1* tumor predisposition syndrome and the possibility to prescribe first-line immunotherapy in countries such as Italy. 

## Figures and Tables

**Table 1:** Significances of BAP1 loss in pleural mesothelioma.

Diagnostic	Predictive	Prognostic
**YES**, loss is indicative of malignancy (100% specificity). BAP1 loss at IHC could likely be used as screening tool for BAP1 tumor predisposition syndrome as instead happens in melanoma	**NO**, although in the Ramucirumab Mesothelioma clinical trial (RAMES), mutation of the gene *BAP1* is related to a prolonged PFS for patients treated with platinum/pemetrexed regimens (*p* = 0.04)	**NO**, limited evidence in the literature

**Table 2:** Tumors implicated in *BAP1* tumor predisposition syndrome [31,32].

*BAP1*-Inactivated Melanocytic Tumors (BIMT; formerly called atypical Spitz tumors), Basal Cell Carcinoma (BCC), Uveal melanoma Meningioma Mesothelioma Renal Cell Carcinoma (RCC) Hepatocellular neoplasms Thymic carcinoma Suspected but unconfirmed tumors in *BAP1*-TPDS include breast cancer, neuroendocrine carcinoma, lung adenocarcinoma, thyroid cancer, and urinary bladder cancer.

**Table 3:** Clinical–pathologic characteristics of patients with pleural mesothelioma and *BAP1* tumor predisposition syndrome [6].

Age	Gender	Histology	Other
Younger than 60 years	Mainly females	Epithelioid, well differentiated with prognostic favorable patterns (tubular and papillary), presence of in situ mesothelioma, multifocal disease, even involving peritoneal and/or pericardium when multiple biopsies are performed	Presence or history of previous tumors. (in particular those linked to *BAP1*-TPDS.)

## Data Availability

No new data were created or analyzed in this study. Data sharing is not applicable to this article.
